# Working memory training restores aberrant brain activity in adult attention‐deficit hyperactivity disorder

**DOI:** 10.1002/hbm.25164

**Published:** 2020-08-19

**Authors:** Juha Salmi, Anna Soveri, Viljami Salmela, Kimmo Alho, Sami Leppämäki, Pekka Tani, Anniina Koski, Susanne M. Jaeggi, Matti Laine

**Affiliations:** ^1^ Department of Neuroscience and Biomedical Engineering Aalto University Espoo Finland; ^2^ Department of Psychology and Speech‐Language Pathology University of Turku Turku Finland; ^3^ Turku Institute for Advanced Studies University of Turku Turku Finland; ^4^ Department of Clinical Medicine University of Turku Turku Finland; ^5^ Department of Psychology and Logopedics University of Helsinki Helsinki Finland; ^6^ AMI Centre, Aalto Neuroimaging Aalto University Espoo Finland; ^7^ Department of Psychiatry Helsinki University Hospital Helsinki Finland; ^8^ School of Education University of California Irvine Irvine California USA; ^9^ Department of Cognitive Sciences University of California Irvine Irvine California USA; ^10^ Department of Psychology Åbo Akademi University Turku Finland; ^11^ Brain and Mind Center University of Turku Turku Finland

**Keywords:** ADHD, brain imaging, cognitive training, working memory

## Abstract

The development of treatments for attention impairments is hampered by limited knowledge about the malleability of underlying neural functions. We conducted the first randomized controlled trial to determine the modulations of brain activity associated with working memory (WM) training in adults with attention‐deficit hyperactivity disorder (ADHD). At baseline, we assessed the aberrant functional brain activity in the n‐back WM task by comparing 44 adults with ADHD with 18 healthy controls using fMRI. Participants with ADHD were then randomized to train on an adaptive dual n‐back task or an active control task. We tested whether WM training elicits redistribution of brain activity as observed in healthy controls, and whether it might further restore aberrant activity related to ADHD. As expected, activity in areas of the default‐mode (DMN), salience (SN), sensory‐motor (SMN), frontoparietal (FPN), and subcortical (SCN) networks was decreased in participants with ADHD at pretest as compared with healthy controls, especially when the cognitive load was high. WM training modulated widespread FPN and SN areas, restoring some of the aberrant activity. Training effects were mainly observed as decreased brain activity during the trained task and increased activity during the untrained task, suggesting different neural mechanisms for trained and transfer tasks.

## Graphical abstract

We conducted the first randomized controlled trial to determine the modulations of brain activity associated with working memory (WM) training in adults with attention‐deficit hyperactivity disorder. WM training modulated widespread areas in the frontoparietal and salience networks, restoring some of the aberrant activity. Training effects were mainly observed as decreased brain activity during the trained task and increased activity during the untrained task, suggesting different neural mechanisms for trained and transfer tasks.

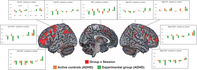

## 1 INTRODUCTION

Adjusting to the massive information flow in the modern society can be a formidable challenge particularly for individuals with attention deficits whose cognitive processes are prone to overload (Klingberg, 2008). The occurrence of attention‐deficit hyperactivity disorder (ADHD) that is typically expressed in difficulties to pay attention, tendencies to act without regard to the consequences, and excessive activity level, has been continuously increasing (e.g., Collins & Cleary, 2016). ADHD is now widely recognized as a multifactorial large‐scale brain connectivity disorder that is coupled with various cognitive impairments (e.g., inhibition, Barkley, 1997; executive function, Boonstra, Oosterlaan, Sergeant, & Buitelaar, 2005; working memory (WM), Alderson, Kasper, Hudec, & Patros, 2013). There is evidence that, at least in some individuals, the symptoms can be remediated and some of the altered brain activity recovered using the gold‐standard stimulant treatments (Biehl et al., 2016; Cubillo et al., 2014; Rubia et al., 2014). Nonetheless, given that stimulant treatments are not effective in up to 30% of the ADHD population (Banaschewski, Roessner, Dittmann, Janardhanan Santosh, & Rothenberger, 2004. Toomey, Sox, Rusinak, & Finkelstein, 2012), alternative and complementary treatment approaches, such as cognitive training, have also gathered considerable interest (for a meta‐analysis, see Cortese et al., 2015).

Many of the proposed cognitive interventions implemented in ADHD populations have been targeting WM. WM involves the conscious maintenance and manipulation of current information, and it is considered as one of the core functions of human cognition (D'Esposito & Postle, 2015). Critically, WM is often impaired in ADHD (e.g., Martinussen, Hayden, Hogg‐Johnson, & Tannock, 2005), with potential downstream effects to other cognitive domains as well (Alderson et al., 2013). While the potential benefits of cognitive interventions in ADHD and its controversies have been widely investigated and discussed in the behavioral domain (Cortese et al., 2015), it remains unclear whether and how cognitive training might influence brain functions in ADHD (Klingberg, 2010).

One of the most widely used WM tasks in clinical research (Jacola et al., 2014), brain imaging (Owen, McMillan, Laird, & Bullmore, 2005; Wang et al., 2019), cognitive training (Au et al., 2015; Soveri et al., 2017b), as well as ADHD studies (see below) is the n‐back task originally developed by Kirchner (1958). The n‐back task is a continuous performance task that requires the participant to decide whether the current stimulus matches the one *n* steps back in the stimulus sequence. Increased load in the task typically reflects decreased hit rates and increased reaction times. Although n‐back performance correlates only weakly with other commonly used WM tasks (Kane, Conway, Miura, & Colflesh, 2007; Redick & Lindsey, 2013), evidence from latent factor analyses has indicated that n‐back tasks are valid indicators of general WM function (Schmiedek, Lövden, & Lindenberger, 2014). Brain areas where the activity is increased together with the increased WM load in the n‐back tasks encompasses the frontoparietal (FPN), salience (SN), sensory‐motor (SMN), and subcortical (SCN) brain networks (Owen et al., 2005; Wang et al., 2019; Yaple & Arsalidou, 2018). These studies have shown a large overlap between the activation patterns associated with n‐back variants with different types of stimuli (e.g., visuospatial, numeric, object). At the same time, different types of n‐back tasks deactivate the default‐mode network (DMN) areas in the posterior cingulate cortex (PCC), precuneus, ventromedial prefrontal cortex (VMPFC), inferior temporal, and lateral occipital/occipito‐parietal cortex (e.g., Pallesen, Brattico, Bailey, Korvenoja, & Gjedde, 2009; see Sonuga‐Barke & Castellanos, 2007 for a review). These activity patterns considerably overlap with those observed in other types of WM tasks (Daniel, Katz, & Robinson, 2016; Emch, von Bastian, & Koch, 2019; Rottschy et al., 2012; Wager & Smith, 2003). Indeed, after more than 1,000 brain imaging studies focusing on WM (see NeuroSynth database for an extensive meta‐analysis), it can now be concluded that detailed process‐wise regional divisions based on the WM component processes are only tentative (see also Bledowski, Kaiser, & Rahm, 2010; Eriksson, Vogel, Lansner, Bergstrom, & Nyberg, 2015; Nee & Jonides, 2008).

In their meta‐analysis of eight experiments, Cortese et al. (2012) reported hypoactivation in the FPN, SN, and SMN in participants with ADHD during performance of WM tasks. Many of the studies included in this meta‐analysis as well as more recent studies relied on n‐back tasks. For instance, in their seminal study, Valera, Faraone, Biederman, Poldrack, and Seidman (2005) reported hypoactivity in cerebellar, occipital and prefrontal areas in twenty participants with ADHD as compared with a similar‐sized control group, and their later study replicated these findings with a larger sample (Valera et al., 2010). Besides these areas, hypoactivation in participants with ADHD has been reported also in the parietal cortex (see for example, Bayerl et al., 2010; Brown et al., 2012; Cubillo et al., 2014; Kobel et al., 2009; Mattfeld et al., 2015). Overall, aberrant activity in the so‐called task‐positive brain networks appears to be a robust neural marker in ADHD, despite the lack of group differences in task performance (e.g., Massat et al., 2012; Valera et al., 2005). ADHD has also often been associated with DMN activity (see Brown et al., 2012 for a study with the n‐back task, and Sonuga‐Barke & Castellanos, 2007, and Bozhilova, Michelini, Kuntsi, & Asherson, 2018 for general reviews). So far, brain imaging research of ADHD has largely focused on studies in children and adolescents, while the adult research remains underexploited (Cortese et al., 2012).

There is evidence that stimulant medication results in restoration effects in WM‐related brain networks in ADHD populations (Biehl et al., 2016; Cubillo et al., 2014; Kobel et al., 2009; Rubia et al., 2014). It has been suggested that the most consistent stimulant‐related recovery occurs in the inferior frontal gyrus (IFG)/Insula (see Rubia et al., 2014 for a meta‐analysis). However, it is still not clear whether and how brain dysfunctions underlying attention deficits respond to cognitive interventions (Olesen, Westerberg, & Klingberg, 2004). In their preliminary study on 18 adolescents with ADHD, Stevens, Gaynor, Bessette, and Pearlson (2016) showed changes in FPN after training on the Sternberg task. However, given the lack of a control group, it remains unclear whether those observed changes were truly training‐related. Recently, de Oliveira Rosa et al. (2019) published another small‐scale pilot study (*n* = 10 for the training group), suggesting that in children with ADHD, computerized cognitive training modulates activity in the insula/putamen and thalamus/pallidus during the n‐back task. As the current knowledge can be considered only preliminary, we set out to perform the first randomized controlled WM training study with an Active control group to examine the malleability of aberrant brain function in adults with ADHD. In healthy adults, practice effects with cognitive tasks are observed both as increases as well as decreases in widespread brain networks (Chein & Schneider, 2005), extended prefrontal activation changes being a unique factor in WM training studies (Salmi et al., 2018). It should be noted, however, that the systematic activation increase/decrease patterns are not well understood (Salmi et al., 2018a), as trained and transfer tasks are not separately assessed in fMRI studies on WM training (but see Dahlin, Neely, Larsson, Backman, & Nyberg, 2008).

Based on the prior WM training studies in healthy participants and ADHD studies using the n‐back paradigm, we formulated three main hypotheses: (a) In healthy participants, prolonged training at capacity limits in ADHD participants results in redistribution (i.e., modulation of the existing WM network) of the task‐related brain networks rather than reconfiguration of the WM‐related brain activity (i.e., recruitment of new areas that are not activated at baseline; for a review and a meta‐analysis, see Constantinidis & Klingberg, 2016, Salmi et al., 2018a). More specifically, based on a recent meta‐analysis of studies performed in healthy adults, we expected that training‐related changes in brain activity would be observed particularly in the dorsolateral prefrontal cortex (DLPFC, Salmi et al., 2018a). (b) We also expected to see restoration of aberrant brain activity that distinguishes participants with ADHD from healthy controls based on previous work (Cortese et al., 2015). (c) Our pre‐post design included trained and untrained variants of the n‐back task, and thus, we aimed to investigate potential differential neural mechanisms for trained tasks and structurally similar near‐transfer tasks. Based on earlier work (see Salmi et al., 2018a), we expected to observe differential training effects in trained task and untrained task given that cognitive skill learning elicited by training tends to be limited to the trained tasks (Bhandari & Badre, 2018). We also collected behavioral data to monitor training progress and its effects on pre‐post behavioral measures, and we expected to see a similar near‐transfer pattern as typically observed in healthy adults (see Soveri et al., 2017b).

## 2 MATERIALS AND METHODS

### 2.1 Participants

Forty‐four individuals with ADHD and 18 healthy controls participated in this study (see Table 1). The participants with ADHD were recruited at the Neuropsychiatry outpatient clinic in the Helsinki University Central Hospital and at two private clinics in the Helsinki metropolitan area (Diacor Healthcare Services in Helsinki and ProNeuron in Espoo). All patients were pre‐screened at the clinic. The healthy controls were recruited mainly via email lists at vocational schools, polytechnics, and universities, and via personal contacts of the authors. Predefined target for the sample size of the training study was 40 participants (20 per condition). The sample size was deemed as sufficient based on previous fMRI studies with cognitive training intervention (Salmi et al., 2018a). Participants had to be native Finnish speakers, have normal or corrected‐to‐normal vision, sufficient hearing, and meet the eligibility criteria for MRI. They were excluded if they had any other severe psychiatric or neurological disorders than ADHD including head trauma demanding treatment, substance abuse or other addictions. The psychiatrists recruiting the participants with ADHD used the Structured Clinical Interview for DSM‐IV Axis I Disorders (SCID‐I) and Mini‐International Neuropsychiatric Interview (M.I.N.I.) to exclude comorbid disorders as part of their regular clinical assessment. The study was reviewed and approved by the Ethics Committee for Gynecology and Obstetrics, Pediatrics and Psychiatry of the Helsinki University Hospital. All participants gave their informed consent according to the Declaration of Helsinki. The participants were reimbursed with 60 € if they participated only to the first measurement, or 240 € if they also completed the intervention and posttest.

**TABLE 1:** Demographics, symptoms, and task performances in (untrained) ADHD participants and healthy controls

Variable		ADHD (*n* = 39)	Healthy controls (*n* = 18)	*p*
Demographics				
Age	Years	28.6 (5.4)	29.61 (8.2)	.65
Education	Level	4.7 (2.2)	6.4 (1.8)	.02
Verbal skills (WAIS vocabulary test)	Standard score	11.1 (2.6)	11.7 (1.8)	.34
Non‐verbal skills (WAIS matrix reasoning)	Standard score	12.3 (2.9)	13.6 (2.2)	.11
ADHD screening tests				
ASRS‐A	Sum score	14.9 (3.7)	7.2 (3.3)	<.001
ASRS‐B	Sum score	27.2 (7.9)	14.5 (6.5)	<.001
ASRS total	Sum score	42.1 (10.8)	21.7 (9.4)	<.001
BRIEF	Sum score	75.8 (20.3)	32.2 (18.0)	<.001
Cognitive tasks				
Dual n‐back	Max level	2.2 (0.6)	2.1 (0.6)	.24
Single n‐back—spatial	Hitrate (%)	89 (12)	89 (8)	.67
Single n‐back—verbal	Hitrate (%)	89 (12)	90 (8)	.60
Running memory—spatial	Lists correct	3.2 (2.1)	4.2 (2.0)	.09
Running memory—verbal	Lists correct	3.3 (1.7)	3.3 (1.9)	.20
Digit span	Sum score	11.3 (3.0)	13.7 (3.0)	<.01
CPT—Errors of omission	Error score	2.2 (3.8)	1.28 (1.6)	.23
CPT—Errors of commission	Error score	17.1 (7.2)	9.3 (4.7)	<.001

*Note: p* represents the group difference. In education level, 0 is no degree, 4 is high‐school, and 9 is PhD.

ADHD was diagnosed according to the Diagnostic and Statistical Manual of Mental Disorders, Fourth Edition (DSM‐IV). In addition to the original diagnostic interview, we conducted the Conners' Adult ADHD Diagnostic Interview for DSM‐IV to confirm the current status (Epstein, Johnson, & Conners, 2001). The patients met criteria for either only inattention or both inattention and hyperactivity. Of the included participants, three had migraine, one had hypothryroidism, and two had experienced mild epilepsy symptoms in childhood but with no treatment needed since that time. In addition to stimulants (*N* = 35), three had been prescribed medication for migraine (two of them use the medicine based on the need and one had prohylactic treatment), one for mild depression (selective serotonin re‐uptake inhibitor, regular use), and one for hypothyroidism (regular use). Out of the participants that had been prescribed stimulants, seven mentioned that they are changing the dose depending on the need. Moreover, eight of the participants that had been prescribed stimulants reported that they are not using the medicine every day or may sometimes take breaks (e.g., during the holidays). One participant reported that currently he/she does not use the stimulants at all, even though he/she has received the prescription. Out of the stimulant users, seven have had this medicine for less than a year. Participants using stimulants had a 24‐hr wash‐out prior to the pre‐post fMRI sessions. Two participants dropped out from the study after the pretest, and three during the training period due to scheduling difficulties (two from the Experimental group and one from the Active control group). The data of one additional participant from the Active control group had to be excluded due to poor quality MRI. Participants with ADHD who were on stimulant medication continued their treatment during the training intervention. The Experimental group and the Active controls did not differ with respect to their stimulant medication (18/20 participants in the Experimental group and 17/18 Active controls were taking stimulants). The healthy controls served as controls only for the pretest, thus being used to test for potential group differences in WM‐related activity.

### 2.2 Self‐ratings

Adult ADHD Self‐Report Scale (ASRS) and Behavior Rating Inventory of Executive Function (BRIEF) adult version were used to self‐rate the ADHD symptoms and related everyday attention deficits, respectively (Table 1).

### 2.3 Cognitive measures

The computerized test battery lasted approximately one and a half hours (see Table 1). The healthy controls completed the test battery once, and the participants with ADHD completed the tasks before and after the training period.

#### 2.3.1 Dual n‐back task

Due to its wide use in brain imaging, cognitive training, and ADHD research (see Section 1), we selected the n‐back task as our training task, as well as an outcome measure before and after training (Jaeggi, Buschkuehl, Jonides, & Perrig, 2008). Despite that the behavioral training and transfer outcomes appear to be quite similar regardless of whether participants train on single or dual n‐back tasks (Au et al., 2015), we selected a dual task for two reasons. First, we wanted to test whether the behavioral training results were similar to our previous dual n‐back training study in a neurotypical population (Soveri et al., 2017a). Second, we assumed that the dual n‐back training would require more widespread cognitive and neural processes as compared to single n‐back training, thus resulting in more extensive changes in brain activity (see Thompson, Waskom, & Gabrieli, 2016). In the dual n‐back task, participants were presented with simultaneous phonological and visuospatial stimulus streams. The phonological stream included eight different spoken Finnish syllables (/dy/, /ki/, /le/, /nä/, /pö/, /ro/, /su/, or /ta/) while the visuospatial stimuli were white squares appearing in eight possible locations on the screen (at top middle, bottom middle, or any of the four left or right corners from the fixation that was at the center). Syllables and squares were presented at the same time (500 ms stimulus presentation, 2,500 ms inter‐stimulus interval). The task was to indicate whether the current stimulus matched the one presented n‐trials back. Participants were required to respond to both stimulus streams, that is, they had to press the right key if the syllable matched the one presented *n* trials back, and the left button if the spatial location of the square matched with the square location *n* trials back. On each trial, a target could appear in the phonological or visuospatial stream, or both. The task consisted of 10 blocks of sequences, each containing 20 squares and 20 syllables. Total duration of the dual n‐back task in the pretest session was about 12 min. The task was adaptive, that is, the difficulty was continuously adjusted according to individual's performance. When 90% accuracy was reached, *n* was increased by 1 in the next block. If the accuracy rate fell below 75% on either stimulus type, *n* was decreased by 1. The level of *n* could vary between 1 and 9. The session began with a 2‐back sequence but changed to 1‐back if the accuracy fell below 75%. At the end of the session, a result screen was displayed showing the highest level of *n* achieved during the session and the number of blocks completed for each level of *n*. Given that the task difficulty varied as a function of each participant's task performance (meaning that accuracy or RT metrics are not comparable across participants), we used the maximum n‐back level achieved per session as the main dependent variable (cf. Soveri et al., 2017b).

#### 2.3.2 Single n‐back tasks

Given that the transfer effects are considered to be relatively narrow (Kirchner, 1958; Melby‐Lervag, Redick, & Hulme, 2016; Soveri et al., 2017b), we used two single n‐back variants of the trained dual n‐back task as separate outcome measures during the pre‐post fMRI sessions, namely a version with visuospatial material (locations) and another one with visually presented digits. In the visuospatial task, white squares were presented in eight locations. In the numerical variant, the stimuli were digits from 1 to 9 presented at the center of the screen. The blocks began with an instruction of the task type (n‐back level). After the instruction, each location/digit remained on the screen for 1,500 ms, with an interstimulus interval (ISI) of 450 ms. Both task variants included four different n‐back levels (0‐back to 3‐back) and the blocks for each n‐back level presented in counterbalanced order (0‐back, 1‐back, 2‐back, 3‐back, 3‐back, 2‐back, 1‐back, 0‐back, etc.). Other than the training task, the single n‐back tasks were not adaptive and two response buttons (match and non‐match) in load levels higher than 0‐back were used. In the 0‐back task, the participants pressed the left button for each stimulus. There were 10 trials requiring a response in each block, of which two trials were match trials (targets) on average. There were five blocks per load level for both digit and visuospatial tasks, and thus, the single n‐back task lasted about 13 min. Accuracy rates (proportion of hits) averaged across the 1‐back, 2‐back, and 3‐back levels were used as the dependent variables. The single n‐back tasks were performed only during the fMRI experiment.

#### 2.3.3 Digit and visuospatial running memory

Participants were presented with sequences of digits or visuospatial stimuli of varying length (Pollack, Johnson, & Knaff, 1959). In the verbal version, the sequences consisted of digits (1–9), and in the visuospatial version, the stimuli consisted of squares appearing at eight different locations. Each cross was visible in the matrix for 1,500 ms. The inter‐stimulus‐interval was 500 ms, during which the matrix was empty. The stimuli were pseudo‐randomized into 28 sequences. The sequence length varied from seven to fourteen, with the exception of two four‐unit catch sequences. Each time a sequence ended, the participants repeated the digits or squares by clicking on corresponding numbers in the screen in the correct order, or by clicking on the correct locations with the mouse. In both tasks, the participants did not know the sequence length beforehand. The dependent variables were the total number of correctly recalled location/digit sequences.

#### 2.3.4 Digit span

In the digit span task, participants were instructed to repeat sequences of digits in the same (forward) or reversed (backward) order (Blankenship, 1938). Sequence length varied between 2 and 9 digits in the forward tasks, and 2 to 8 digits in the backward tasks. Digits were presented with the interval of one per second. Two sequences of each length were administered. When the participant answered correctly to one of the sequences, the sequence length was increased by one digit. The task ended once the participant made two errors in a row. The dependent variable was the total number of correctly reported sequences (averaged across the forward and backward versions).

#### 2.3.5 Continuous performance test

In the continuous performance test (CPT) task, participants were presented with a sequence of letters with fixed alternating intervals (1,000, 2000, and 3,000 ms) (Rosvold, Mirsky, Sarason, Bransome, & Beck, 1956). They were required to press the space bar for each letter, except for the letter X (probability 9.7%). There were 360 trials, and the duration of the task was approximately 14 min. Two dependent variables were used, omission errors as a proxy for inattention, and commission errors, as a proxy for impulsivity.

### 2.4 Intervention procedure

**FIGURE 1:**
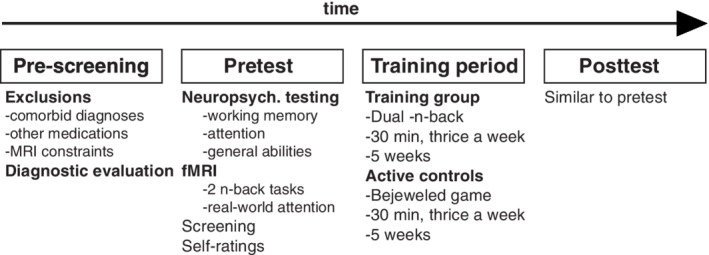
Illustration of the main aspects at different stages of the study

The training period lasted for 5 weeks (cf. Figure 1, Figure S1, and Soveri et al., 2017a for a similar behavioral study in healthy adults). Participants in both intervention groups trained three times a week, once in the laboratory (Department of Psychology, University of Helsinki) and twice at home. A minimum of 10 sessions were required to be invited to the posttest, and the time spent on training was the same in both groups (~25 min per session). Pre/posttest sessions and training were supervised by different experimenters. A lottery‐based randomization (20 tickets for the Experimental group and 20 for the Active controls) was performed individually for each participant after the pretest by the person who was about to supervise the training session. Thus, the experimenters conducting the assessments as well as the imaging sessions were blind with respect to the group membership of the participant. The participants themselves were also unaware of whether they were in the Experimental group or Active control group. Participants received a laptop computer to conduct the practice at home, but they were also allowed to use their own computer.

#### 2.4.1 Experimental group

The participants in the Experimental group were trained on the dual n‐back task that was the same as the one used in the assessment battery, except that the number of blocks in each training session was 20 instead of 10. After each training session, participants completed a questionnaire in which they indicated the maximum *n*‐level they had achieved, as well as their level of motivation and arousal during the training session using a 10‐point Likert scale (where 0 = very low and 10 = very high). In addition, the program generated a data logfile that was stored on the computer. Each participant performed at least 11 training sessions, and 11 participants completed all 15 training sessions (mean number of sessions = 13.8, *SD* = 1.7, see Figure S2).

#### 2.4.2 Active control group

The Active controls played the “Bejeweled II” video game by PopCap Games. In this 2004 computer game, the task is to score points by swapping a jewel with an adjacent one to create a chain of jewels of the same color. The game for the Active controls was selected based on its limited demands on WM, general appeal to a wide audience, and its successful use in prior studies (e.g., Soveri et al., 2017a). Participants played the game for about 25 min in every training session, and they also recorded their highest scores in personal training logs. Everyone completed at least 11 sessions, and 15 participants completed all 15 training sessions (mean number of sessions = 14.6 [*SD* = 1.0], see Figure S2).

### 2.5 Behavioral analyses

Baseline differences comparing the healthy controls and participants with ADHD, as well as between the two training groups consisting of participants with ADHD, were assessed using independent samples *t*‐test and analyses of variance (ANOVA). Within the trained group, we tested for changes in performance or symptom scores from pretest to posttest using paired‐samples t‐tests. Furthermore, the effects of training were examined using analyses of covariance with posttest performance as the dependent variable, intervention group as fixed factor, and pretest performance as the covariate. As the influence of dual n‐back training on symptoms is shown to be associated with training progress (Jones, Katz, Buschkuehl, Jaeggi, & Shah, 2018), the participants in the Experimental group were divided into high‐gainers and low‐gainers based on a median split on their dual n‐back improvement from pretest to posttest to examine the effect of training on symptoms.

### 2.6 MRI acquisition

We collected fMRI data at Advanced Magnetic Imaging Centre (Aalto University) using a Siemens MAGNETOM Skyra 3 T scanner (Siemens Healthcare, Erlangen, Germany) which was mounted with a 30‐channel head coil. We conducted two functional runs using a gradient‐echo echo planar imaging sequence. In fMRI, the following imaging parameters were used: TR 1.9 s, voxel matrix 64 × 64, slice thickness 3.0 mm, in‐plane resolution 3.1 mm × 3.1 mm × 3.0 mm. Timing of the fMRI scanning was random in relation to the presentation of the stimuli, and the first four volumes in each image time series were discarded to stabilize magnetization. Functional measurements consisted of 816 volumes total (408 for the visuospatial and digit n‐back tasks). Besides fMRI, a structural MR image with a T1‐weighted MPRAGE sequence (TR 2.5 s, voxel matrix 256 × 256, slice thickness 1 mm) was acquired before the third functional run for registration purposes. The participants did not report any considerable harms following the MRI.

The tasks presented during fMRI experiment were projected on a semitransparent screen behind the participants' head using a 3‐micromirror data projector (Christie X3, Christie Digital Systems, Mönchengladbach, Germany). The distance to the screen was approximately 34 cm via a mirror located above the eyes of the participant (binocular field of view 24 cm).

### 2.7 fMRI analyses

The fMRI data were analyzed using FSL tools (Smith et al., 2004). Motion correction was performed using FMRIB's Linear Image Registration Tool (MCFLIRT). We used the Brain Extraction Tool (BET) for T1 as well as functional images to isolate the brain tissue from the non‐brain tissue. The linear registration of the functional image via the anatomical image to standard space (MNI152 template, Montreal Neurological Institute) was performed using FMRIB's Linear Image Registration Tool (FLIRT). The registration of the functional image to the anatomical image was performed using six rigid body transformations. In the linear transformation from anatomical to standard image, we used 12 degrees of freedom. Residual motion was regressed from the functional data in the modeling stage. We also confirmed that there were no group differences in mean displacements in any comparison (see Table S1) and that there were no clear spikes in the data included in the analysis. The functional data were high‐pass filtered using a 100‐s cutoff. Spatial smoothing was performed separately on each volume of the data by setting a 4 mm Gaussian kernel to the signal.

We performed general linear model (GLM) data‐analyses using fMRI Expert Analysis Tool software (FEAT, Woolrich, Ripley, Brady, & Smith, 2001), Functional Magnetic Resonance Imaging of the Brain Centre (FMRIB) software library (FSL, release 5.0.9). FMRIB's Improved Linear Model was used in the first level analysis. Gamma function was used in the convolution of the hemodynamic response function. First‐level standard GLM included three task regressors (one each for 1‐back, 2‐back, and 3‐back tasks), and nuisance regressors for instructions (1) and motion (6). The 0‐back task (press a yes/no button to each stimulus) was used as a baseline in the model. The resulting first level contrasts were 1‐back versus 0‐back, 2‐back versus 0‐back, and 3‐back versus 0‐back. This analysis was conducted to reveal the activations at different load levels. Comparisons to the 0‐back task were selected instead of load effects (2‐back vs. 1‐back, 3‐back vs. 2‐back, and 3‐back vs. 1‐back), since the effects of load are not linear and therefore the training effects as well as the group differences would be more difficult to interpret. In addition, we determined the activations (1 1 1) and deactivations (−1 –1 −1) across the three load levels. At the first level, the data for visuospatial and digit single n‐back tasks were analyzed separately. The same high‐pass filter was used for the model and time series data.

**FIGURE 2:**
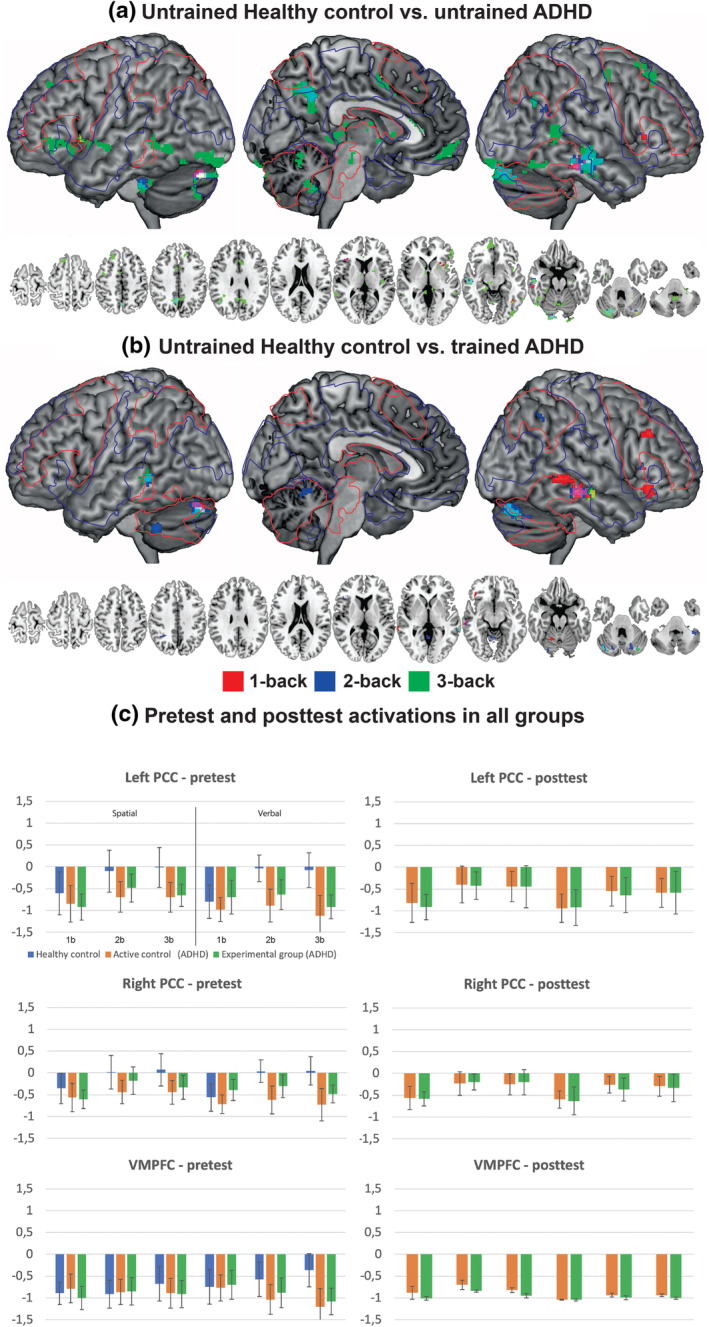
(a) Group differences (healthy controls vs. participants with ADHD) in brain activity in untrained participants during the n‐back tasks at each load level (1‐back vs. 0‐back, 2‐back vs. 0‐back, and 3‐back vs. 0‐back). There was no significant brain activity in the opposite contrasts (participants with ADHD vs. healthy controls). (b) Brain activity of the participants with ADHD in the Experimental group at posttest as compared with the untrained healthy controls. Red and blue colored edges in each brain rendering shows the borders of the activations and deactivations across all participants and load levels. (c) Training effects in the Experimental group and healthy controls in DMN hubs. *Z* = 3.5, corrected *p* < .05

Second‐level analyses examining the group comparisons and training effects were conducted using FMRIB's Local Analysis of Mixed Effects (FLAME, Woolrich, Behrens, Beckmann, Jenkinson, & Smith, 2004). This method was chosen instead of non‐parametric permutation inference (Randomize), as permutation testing is neither validated nor recommended for multiway ANOVAs with three factors (for discussion on permutation testing with ANOVAs, see Manly & Francis, 1999, Francis & Manly, 2001). We conducted a second‐level analysis comparing the activations for the visuospatial and digit single n‐back tasks (see Figure S3). However, in further analyses, we combined the two tasks to maximize the statistical power. The first‐level contrasts revealing the activations at different load levels (combining the data from the visuospatial and digit tasks) were compared across the ADHD and healthy control groups (1 –1 and −1 1). Activations and deactivations across all participants (see overlays in Figures 2 and 4) were analyzed across all participants to interpret the group differences and training effects. To determine the training effects in the ADHD group, we conducted a mixed ANOVA with factors Session (pretest, posttest), Group (Experimental group, Active control group) and Task (visuospatial task, digit task), with a particular interest on the Group × Session interaction. We also compared trained the participants with ADHD (both groups separately) and the untrained healthy controls to determine whether the aberrant brain activity is recovered during the training (similar to Stevens et al., 2016). To interpret the training effects and possible recovery of aberrant activity, we separately plotted the mean amplitudes in regions of interest (10 mm radius) based on the voxels showing peak activations (see Figure S4). In the whole brain analyses, cluster‐based thresholding was used to account for multiple comparisons. The reliability of FLAME was confirmed by Eklund, Nichols, and Knutsson (2016). To make sure that our analyses do not contain false positives, we used relatively high threshold values (*Z* > 3.5, *p* < .05).

## 3 RESULTS

### 3.1 Behavioral performance at baseline (ADHD participants vs. healthy controls)

Task performance comparing participants with ADHD and healthy controls at baseline are reported in Table 1. The only significant group differences were observed in the CPT and digit span WM task in which the participants with ADHD performed worse than the healthy controls. In the visuospatial and number single n‐back tasks performed during the fMRI session, both groups showed the canonical load effect (see Table S2). The visuospatial and number tasks did not differ significantly in difficulty (Main effect of Condition: (*F*[1,40] = 0.02, *p* = .97, *η*_p_^2^ = 0.00).

### 3.2 Brain activity in the n‐back tasks at baseline (ADHD vs. healthy controls)

The analysis across all participants and task loads showed expected activations in FPN, SN, inferior temporal/lateral occipital cortex, and several subcortical areas, including the cerebellum and striatum. The opposite contrast revealed deactivations in DMN areas in the PCC/precuneus, VMPFC, SMN, and inferior temporal, and lateral occipital/occipito‐parietal cortices (Figure 2a). Brain activity was largely similar between the visuospatial and digit n‐back tasks, mirroring the behavioral data. The only significant stimulus‐type effects across all participants were observed in the dorsal visual, mainly in the lateral occipital cortex (Figure S3), bilaterally. Due to the large overlap between the two single n‐back tasks, we collapsed the data across both task variants for the subsequent analyses to increase power.

In the 1‐back versus 0‐back tasks, group differences (healthy control > ADHD) were observed in the bilateral IFG, left posterior cerebellum, and right medial temporal gyrus (Figure 2a, Table 2). In the 2‐back versus 0‐back tasks, there were additional group differences in the activity of the right inferior parietal cortex, precuneus/PCC, right posterior cerebellum, and left anterior cerebellum (Figure 2a, Table 2). In addition to areas showing group differences in the 2‐back versus 0‐back tasks (excluding right IFG that showed group difference only in the 1‐back vs. 0‐back contrast), the 3‐back versus 0‐back tasks also revealed group differences in the VMPFC, right SMN, SMA/ACC, the bilateral visual cortices, and several subcortical areas (Figure 2a, Table 2).

**TABLE 2:** Labels of brain areas activated in the single n‐back tasks in healthy controls versus ADHD participants at baseline

	Voxels	*Z*‐score	*x*	*y*	*z*
2‐back versus 0‐back					
Right middle temporal gyrus	294	5.35	58	−14	−18
Right posterior cerebellum	280	6.78	24	−82	−26
Right precuneus	211	5.36	12	−60	38
3‐back versus 0‐back					
Right posterior cerebellum	451	6.03	26	−82	−28
Left precuneus cortex	407	6.04	−12	−56	32
Right middle temporal gyrus	326	4.91	52	−28	−16
Left posterior cerebellum	325	4.89	−28	−76	−44
Right medial frontal gyrus	166	4.9	12	54	−8
Right parahippocampal cortex	126	4.79	16	−32	−6
Right inferior frontal gyrus	117	4.88	−50	30	−2
Right anterior cingulate gyrus	109	4.28	6	4	42
Right parahippocampal gyrus	109	4.86	20	−6	−26
Vermis	104	4.86	2	−56	−34

*Note:* For each area, we report numbers of voxels, *Z*‐scores, and maximum coordinates for each activation cluster above 100 voxels.

### 3.3 The effects of training on task performance in adults with ADHD

Experimental group and Active controls did not differ with regard to their demographics or attention symptoms at pretest (Table S3). Across the training period, participants' self‐reported motivation in the Experimental group was 7 on average (*SD* = 1.8) and their arousal level was at 5.8 (1.8) (see Figure S2). At training start, participants were relatively optimistic by indicating that the training might have benefits with regard to their ADHD symptoms (mean score = 8.0 [1.0] on a scale from 1 to 10, with 10 corresponding to highly confident). The Active controls reported an average motivation level of 6.3 (*SD* = 2.2) and an arousal level of 6.4 (*SD* = 2.0) (cf. Figure S2). Presumably due to the high attentional demands, even the control group expected some training benefits with regard to their ADHD symptoms at the beginning of training (mean score = 6.6; *SD* = 2.5). There were no differences between the Experimental and Active control groups in reported motivation (*t* = 0.91, *p* = .37) or arousal levels (*t* = 0.91, *p* = .37), and furthermore, there were no significant group differences in expected training benefits between the two groups (*t* = 1.91, *p* = .07).

**FIGURE 3:**
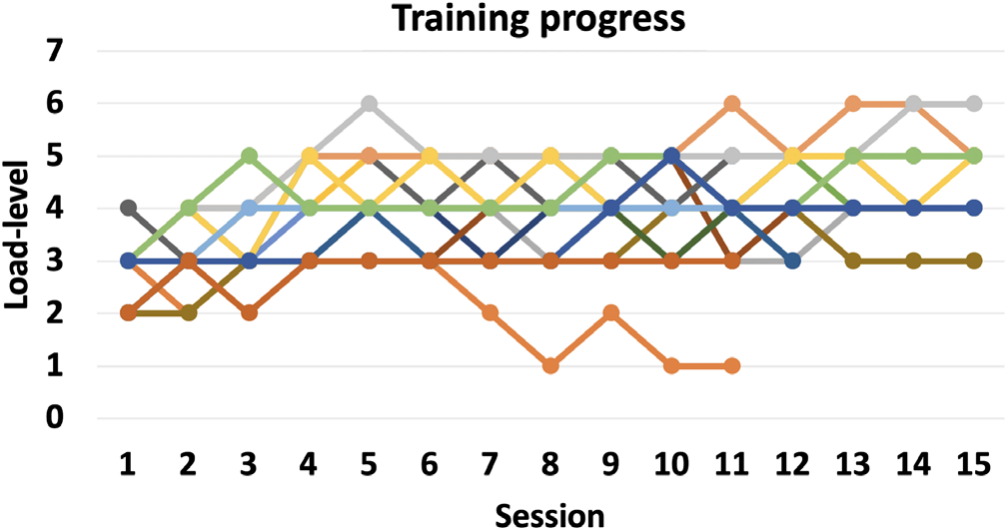
Maximum n‐level in the dual n‐back task in each training session (1–15) for each participant with ADHD in the Experimental group

**FIGURE 4:**
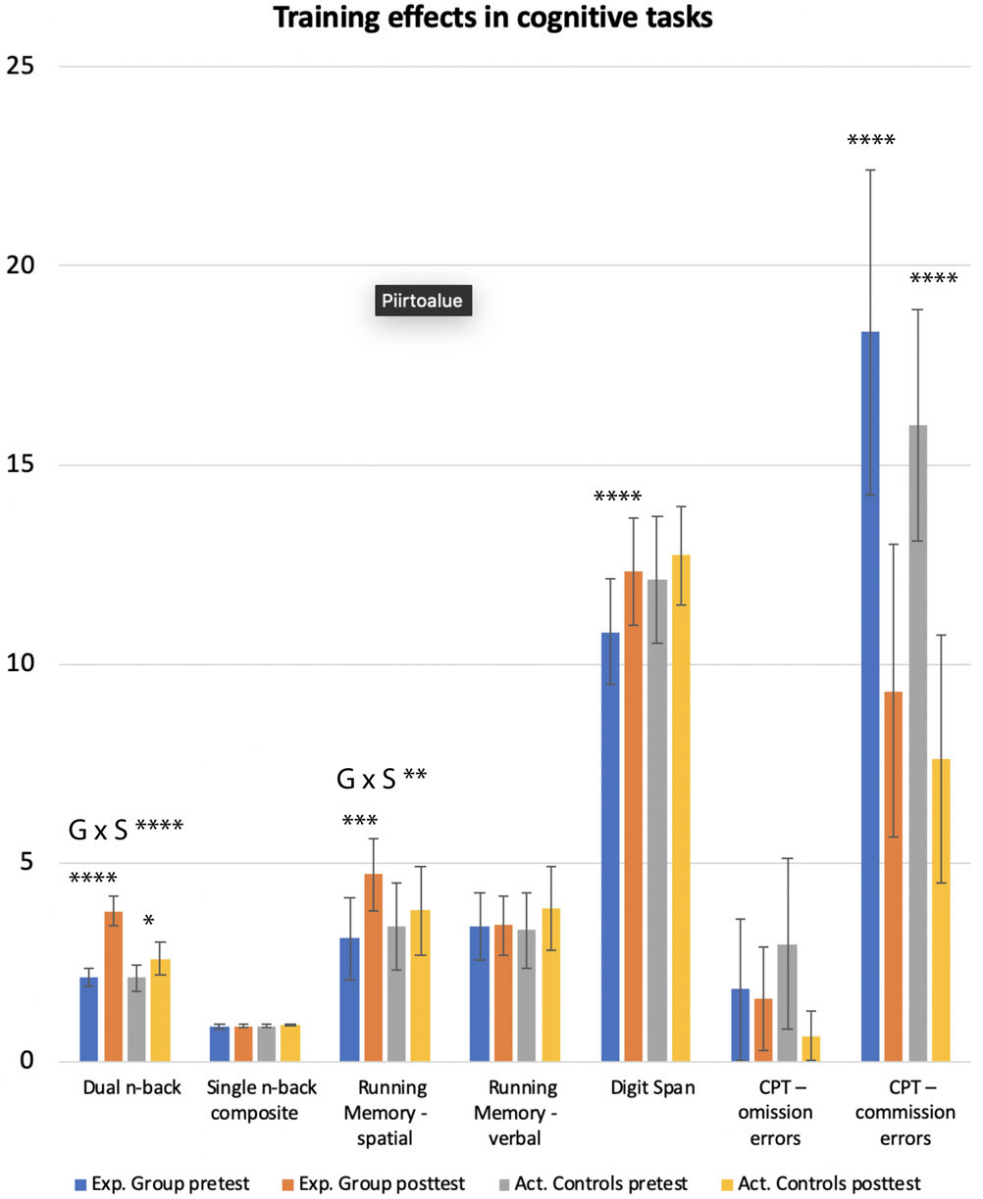
Training effects in the Experimental group and Active control group. G × S refers to Group × Session interaction

The participants with ADHD assigned to the Experimental group significantly improved their performance in the dual n‐back task as a function of training (Figures 3 and 4, Table S4). Although the participants with ADHD assigned to the Active control group showed improved performance in the dual n‐back task at posttest as well, the improvement was more pronounced in the Experimental group (Group × Session interaction (*F*[1,38] = 28.3, *p* < .0001, *η*_p_^2^ = 0.45). In contrast, in the single n‐back tasks performed in the fMRI scanner, there were no significant pre‐ versus posttest changes within either group, nor were the Group × Session interactions significant (Table S4).

The Experimental group also improved more than the Active control group in the visuospatial version of the running memory task (Group × Session interaction (*F*[1,38] = 6.9, *p* < .05, *η*_p_^2^ = 0.17), but not in the digit version (Figure 4, Table S4). In the digit span task, only the Experimental group improved their performance from the pretest to posttest, but the Group × Session interaction was not significant (Figure 4, Table S4). In CPT, both groups made fewer Commission errors at posttest, and in addition, the Active controls made fewer Omission errors (Figure 4, Table S4). However, none of the Group × Session interactions were significant.

The symptom scores in ASRS‐B and the total ASRS scores numerically decreased in the Experimental group at posttest (Table S4). However, neither of these effects nor the Group × Session interactions were significant. In the ASRS‐A scores, there were no changes within or across the two groups. Nonetheless, in order to further explore any training‐related changes on symptoms, we conducted a median‐split within the Experimental group separating high and low gainers according to their dual n‐back improvement from pretest to posttest. This analysis revealed that participants in the Experimental group who improved less showed a significant decrease in their ASRS‐A (Group × Session *F*[1,20] = 5.8, *p* < .05, *η*_p_^2^ = 0.26), ASRS‐B (Group × Session *F*[1,20] = 7.3, *p* < .05, *η*_p_^2^ = 0.30), and in ASRS‐total scores (Group × Session *F*[1,20] = 7.7, *p* < .05, *η*_p_^2^ = 0.31) relative to Active controls.

### 3.4 The effects of training on WM‐related brain activity in adults with ADHD

**FIGURE 5:**
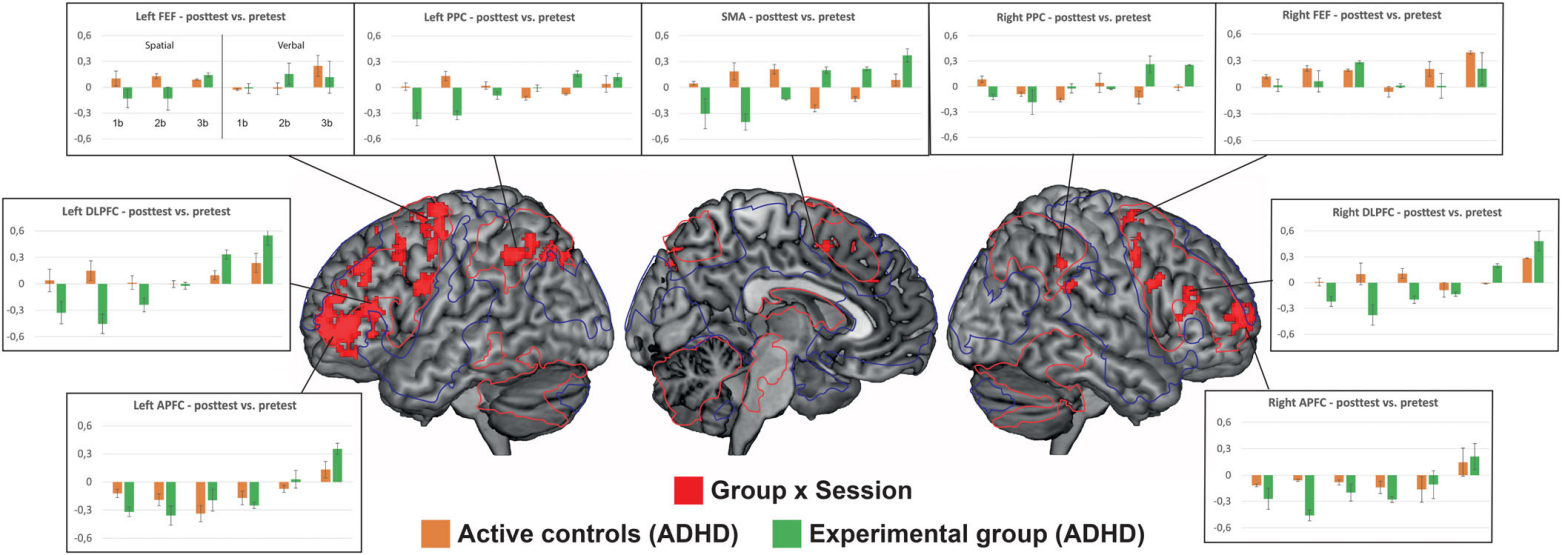
Training‐related modulations (Group [Experimental group, Active control group] × Session [pretest, posttest] interaction) of brain activity in the n‐back tasks in participants with ADHD. *Z* = 3.5, corrected *p* < .05. We separately plotted the effects in each individual condition in key regions of interest to clarify the interpretation of the results

When the changes in the Active control group were accounted for (Group × Session interaction, Figure 5, Table 3), we observed training‐related brain activation changes only in the analysis that was performed across each n‐back load level. This analysis revealed Group × Session interactions in widespread areas covering the DLPFC, superior and inferior parietal cortex, precuneus, and SMA/ACC. Plotting the data separately between spatial and digit tasks suggested different patterns for trained task and untrained task (Figure 5). More specifically, in the trained task, the activity in the Experimental group was typically decreased, while in the untrained task the activity was increased. Hence, there were two distinct patterns of training effects that contributed to the Group × Session interaction.

**TABLE 3:** Labels of brain areas showing training effects in ADHD participants (group × session interaction) across all load levels, and numbers of voxels, *Z*‐scores, and maximum coordinates for each activation cluster above 100 voxels

	Voxels	*Z*‐score	*x*	*y*	*z*
Left frontal pole	1,190	6.26	−28	62	4
Left middle frontal gyrus	378	5.59	−30	−6	56
Right precentral gyrus	334	5.21	36	−62	34
Right frontal pole	273	5.38	30	60	12
Right middle temporal gyrus	265	5.19	34	2	48
Right superior frontal gyrus	227	4.92	22	0	62
Right inferior frontal gyrus	167	5.31	38	32	14
Left precuneus	127	4.75	−8	−74	34
Right paracingulate gyrus	120	4.87	10	38	26
Left supramarginal gyrus	112	5.01	−48	−50	44
Left lateral occipital gyrus	101	5.8	−22	−72	42

As compared with the healthy controls at baseline, the brain activity in the Experimental group at post‐test differed in the 1‐back versus 0‐back task mainly in right DLPFC, IFG, and MTG, and in the left posterior cerebellum (Figure 2b). In the 2‐back versus 0‐back task, differences between the Experimental group at post‐test and healthy controls at baseline were observed in right IFG, PPC, bilateral MTG, left anterior and posterior cerebellum, and right posterior cerebellum. After training, Experimental group differed from healthy controls only in the bilateral MTG and left posterior cerebellum. Thus, the group differences in DMN (precuneus/cuneus and VMPFC) observed prior to the training period were no longer present after the training period, especially in the demanding 3‐back versus 0‐back condition. However, plotting the data in areas showing peak activations (see Figure S4) suggested similar pre‐post effects in the Experimental group and Active control group (Figure 2c).

## 4 DISCUSSION

We conducted the first randomized controlled trial to reveal how the brain functions in adult ADHD might respond to a targeted WM intervention. We first compared brain activity during n‐back WM tasks between untrained participants with ADHD versus healthy controls to examine the aberrant brain activity. Then we randomly assigned the participants with ADHD to one of two interventions: an Experimental group who trained thrice a week on a dual n‐back task, and an Active control group who trained on an object matching game (Bejeweled 2) with lower WM demands.

### 4.1 Baseline differences between ADHD versus healthy controls

#### 4.1.1 Cognitive functions

As expected, as compared with healthy controls, participants with ADHD exhibited a range of deficits, ranging from self‐rated clinical symptoms in inattention and impulsivity to related behavioral performance as indicated by CPT and digit span at baseline (Table 1). Those effects are consistent with previous ADHD studies (see Corkum & Siegel, 1993; Martinussen et al., 2005). Also consistent with other work is our observation that adults with ADHD performed relatively well on n‐back tasks, showing similar behavioral performance levels as the healthy controls (e.g., Massat et al., 2012; Valera et al., 2005).

#### 4.1.2 WM‐related brain functioning

Consistent with our hypotheses, as compared to healthy controls, our ADHD participants displayed aberrant WM‐related brain functioning at baseline. These group differences were observed as decreased brain activity in the ADHD group in the DMN, SN, FPN, as well in the occipital and temporal cortex, and cerebellum, relative to healthy controls (Figure 2a, Table 2). These brain areas have been frequently implicated in prior n‐back studies using participants with ADHD (Bayerl et al., 2010; Brown et al., 2012; Cubillo et al., 2014; Kobel et al., 2009; Mattfeld et al., 2015; Valera et al., 2010). Those effects seem to be consistent despite the often observed lack of group differences in task performance (Massat et al., 2012, Valera et al., 2005), using other WM tasks (Cortese et al., 2012), or even at resting state (Castellanos & Proal, 2012). Our study adds to that literature in that our group differences in brain activity were observed despite comparable behavioral performance, and thus, the effects cannot be explained by the task being more difficult for the ADHD participants (Salmi et al., 2018). Importantly, group differences were reflected in more distributed brain networks in conditions with higher WM demands, which is consistent with prior studies.

### 4.2 Training study

#### 4.2.1 Behavioral findings

In our randomized intervention within the participants with ADHD, we observed behavioral effects that are broadly in line with previous WM training studies in ADHD (Cortese et al., 2015) and healthy controls (see Melby‐Lervag et al., 2016; Soveri et al., 2017b). Specifically, the ADHD group that practiced WM with a dual n‐back task improved not only in the trained tasks (Figure 3), but also in some other closely related WM measures, namely in digit span and visuospatial running memory tasks (Table S4). Both groups improved in an untrained attention task (CPT), which distinguished ADHD participants from the healthy controls at pretest (Table S4), indicating that either both interventions required attentional processes, and/or that the findings reflected test–retest effects. However, given that CPT performance is generally stable and test–retest effects are rarely observed (Halperin et al., 1991), these improvements might still be of clinical value, especially since the participants with ADHD did no longer differ from the healthy controls at post‐test (see commission errors at Table S4). The limited effects of WM training on ADHD symptoms in adults are also in line with previous work (Dentz, Guay, Parent, & Romo, 2017). Curiously, self‐reported ADHD symptoms decreased relative to the Active control group only in those participants who improved the least in the trained task. This result could stem from constantly higher attentional effort for those participants that do not manage to develop new strategies (see Fellman et al., 2020). It should be noted that this effect is rather opposite to the “rich get richer” effect that is frequently reported in cognitive training (Guye, De Simoni, & von Bastian, 2017; Karbach, Könen, & Spengler, 2017; Lövdén, Brehmer, Li, & Lindenberger, 2012), but there is also prior evidence for the compensation hypothesis (that those who initially have problems would benefit more is weaker) that we observed here (Lövdén et al., 2012).

#### 4.2.2 Training‐related modulation of WM networks in ADHD

Our findings concur with and extend previous work with adolescent ADHD (Stevens et al., 2016) in important ways. Specifically, here we show that WM training effects on brain networks are observed in adults with ADHD when compared to active controls, and furthermore, using an intervention task (n‐back) that has been widely used in cognitive training studies (Pergher et al., 2020). Thus, our findings are in line the suggestion that functional WM components are shared across specific task types (Daniel et al., 2016; Emch et al., 2019; Rottschy et al., 2012; Wager & Smith, 2003). In contrast, a recent pilot study conducted in children with ADHD did not find any training‐related modulations in the cerebral cortex (de Oliveira Rosa et al., 2019), which could be related to the different age groups and/or their small sample size.

As expected, Intervention (Experimental group vs. Active control group) × Session (Pre vs. Post) interactions were almost exclusively observed in the areas that were already activated at baseline (see Figures 2a,b). Specifically, training‐related modulations of brain activity were observed in widespread brain networks, encompassing several PFC areas covering dorso‐ and ventrolateral areas and extending to the medial areas (SMA), as well as bilateral PPC. The present results concerning the PFC are consistent with a recent meta‐analysis demonstrating that modulation of this region differentiates WM training studies from training studies targeting perceptual‐motor functions (Salmi et al., 2018a). Although training‐related modulations of PPC activity appear not to be as consistently reported in healthy controls than training effects in the PFC (Salmi et al., 2018a), the involvement of the PPC has been suggested in various studies since Olesen et al. (2004), see Constantinidis and Klingberg (2016) for a review). Also, the effects of WM training on PFC activity were perhaps more widespread in the present study than in many other studies. However, all PFC areas showing training‐related modulations in the present study have been reported also in prior human brain imaging studies on WM training. Hence, the widespread effects are probably rather explained by the magnitude of the effect rather than less focal pattern as such (Constantinidis & Klingberg, 2016; Salmi et al., 2018a). There is also evidence of training‐related modulations of functional (Astle, Barnes, Baker, Colclough, & Woolrich, 2015; Jolles, van Buchem, Crone, & Rombouts, 2013; Takeuchi et al., 2013) and anatomical (de Lange et al., 2017; Salminen, Mårtensson, Schubert, & Kühn, 2016; Takeuchi et al., 2010) connectivity in these networks, as well as changes in related neurotransmitter system activity (Bäckman & Nyberg, 2013). With regard to PFC, there is even evidence of training‐related changes in PFC neuronal population coding in non‐human primates (Meyers, Qi, & Constantinidis, 2012). Hence, also with regard to ADHD, a variety of brain imaging techniques could be utilized to characterize the training‐related plasticity with even greater detail.

Brain areas where training effects have been repeatedly demonstrated in prior studies but were not observed in the present study mostly limit to subcortical areas such as the striatum (Bäckman & Nyberg, 2013). There could be several reasons why modulations of the striatum activity were not observed here. Firstly, like in other subcortical areas, striatal response amplitude changes are generally smaller than those in the cerebral cortex. It is possible that our experimental design was simply not sensitive enough to detect such effects. Secondly, it could be that some other metric than amplitude change would be better suited to detect training effects in the striatum. For instance, functional connectivity or response variability might be theoretically motivated here, because of the role of the striatum in modulating activity in the cerebral cortex. Thirdly, the registration was here conducted at the whole brain level, with methods that are not optimized to accurately align subcortical areas. Therefore, it could be that the possible training effects in the striatum may have been blurred by modest registration errors at the group level analysis. Finally, it could simply be that striatal signals are not modulated by training in ADHD participants similarly to healthy participants. Due to the important role of the striatum in ADHD (see Castellanos & Proal, 2012), it would be important to examine this issue further in separate studies targeted to detect modulations of striatal activity.

As expected, in the present study, training effects were observed both as increases and decreases in brain activity (Chein & Schneider, 2005; Salmi et al., 2018). The present study included for the first time both a criterion task and a structurally similar near‐transfer task, showing differential training effects in these two conditions. More specifically, we observed widespread training‐related brain activity decreases during the trained task, and activity increases during the structurally similar task variant. Our finding is consistent with the skill‐learning approach that emphasizes task‐specific learning (Chein & Schneider, 2012). Based on this approach, the differences between the training‐related changes in the trained task and untrained task could be well explained by the different processes involved at different stages of learning. More specifically, in the trained tasks, gradual automatization is likely to have taken place while in untrained tasks, the training could evoke enhanced attentional engagement that is considered to happen at earlier stages of learning (Chein & Schneider, 2012, see also Kuhn et al., 2013). The lack of automatization in the untrained task could also explain why the training effects are often quite short lasting (see Melby‐Lervåg & Hulme, 2013).

#### 4.2.3 Restoration of the aberrant activity in ADHD

Our results revealed that compared to healthy controls, several brain areas indicating aberrant activity in participants with ADHD at baseline showed training‐related restoration after the intervention. Specifically, at post‐test, brain activity patterns in participants with ADHD were no longer different from healthy controls in the bilateral DLPFC, SMA, FEF, and PPC. We interpret those effects as modulations of WM‐related activity as a result of training, in line with the redistribution hypothesis. Previous ADHD studies have not been able to demonstrate such effects given the lack of healthy controls needed to determine the aberrant activity at baseline, and the lack of employment of active controls. Yet, there is some evidence of restoration of brain function beyond task‐related effects in schizophrenia research (Li et al., 2015), and thus, our results are consistent with those earlier findings, reflecting responsivity of brain areas implicated in ADHD to targeted training.

Activity in the DMN hubs (precuneus/PCC, MPFC, and angular gyrus) that distinguished participants with ADHD from the healthy controls at baseline (Figure 2a), indicated similar restoration effects at post‐test. However, as illustrated in Figure 2c, these effects were not restricted to the Experimental training group, but they were also observed in the Active control group. It has been demonstrated that the functioning of these areas is modulated by mindfulness intervention (Bachmann et al., 2018), and there is also some evidence that stimulant treatments affect recovery of DMN functions (Cubillo et al., 2014). Thus, the present findings suggest that extended training with an attention‐demanding task might influence DMN in ADHD participants, but such effects may not be specifically related to WM. The DMN restoration effects seem to be restricted to anterior areas given that neither the dorsal medial DMN (medial/inferior temporal cortex) nor the posterior cerebellum showed any restoration effects after training. It should be noted that monitoring of the activation changes during the training period would be useful to confirm that these effects are not simply test–retest effects.

Evidence for the restoration of aberrant brain functions could have important clinical applications. For example, brain imaging might not only improve our understanding about the malleability of brain functions, but they could also lead to new targets for interventions. For example, brain imaging could, for instance, reveal “bubbling under” effects (changes in the brain that are not yet readily observed in behavior) that could be further examined in behavioral studies. It might also be that the behavioral outcome measures tapping other cognitive functions (Jaeggi & Buschkuehl, 2014) or everyday problems (Cortese et al., 2015) do not always capture the relevant effects. Overall, more work is needed to better understand the potential implications of WM training, especially by using both trained and non‐trained versions of the tasks as outcome measures in order to shed more light on potential differential effects on brain activity.

## 5 LIMITATIONS

One of the key limitations in contemporary cognitive training research is that the transfer effects are often limited (Melby‐Lervag et al., 2016; Soveri et al., 2017a). Although we did observe some effects that generalized beyond the trained tasks, the effects were also modest in our study. Similarly, the neuroimaging effects observed in the present study are not that strong, which might be related to the lack of behavioral effects. It is well known that there are considerable individual differences in training and transfer outcomes (Jaeggi, Buschkuehl, Shah, & Jonides, 2013) and this might be an issue especially in relatively small sample sizes typically gathered in brain imaging studies. The trade‐off in selecting a paradigm that involves a multitude of WM component functions is that the training results should not be interpreted too narrowly in terms of a specialized subfunction. The present group comparisons at pretest and posttest could well be affected by the individual differences in task demands, arousal, and motivation, which we were not able to test in this sample. It is also possible that training improvement was somewhat affected by part of the training being conducted at home. This could cause individual variability that may diminish some of the effects at group‐level. We observed robust training effects on brain activation in single n‐back tasks only when the analysis was performed across the three load levels. Even though it is not possible to use the generally recommended permutation‐based thresholding in this type of ANOVA design, however, this training effect was robust across the three load levels of the n‐back task even with a highly stringent thresholding. Combining multiple load levels in the analysis may, however, limit the interpretation of the findings. For instance, the non‐focal nature of the training effects could relate to less demanding tasks being included to the analysis, even though it has been suggested that WM training does involve also many other brain areas besides DLPFC (Salmi et al., 2018a). In future studies, separating the load effect from the general training effect might be possible by increasing the amount of data collected per each load level or by decreasing the number of difficulty levels. Finally, using unwarping based on field maps, we could have possibly obtained slightly better registration of individual functional brain maps to the standard space, especially at the VMPFC and inferior temporal areas, which might have resulted in some activations that we were not able to reveal with the current analysis.

## 6 CONCLUSIONS

The present study provides the first evidence that ADHD brain functions are still responsive to WM training at adulthood. We also corroborate the preliminary results on adolescents with ADHD reported by Stevens et al. (2016), suggesting that brain functions in ADHD can be modulated by extensive cognitive training. Practicing WM with a dual n‐back task resulted in modulations of task‐positive FPN activity, specifically, WM training resulted in a redistribution of WM‐related activity which was most prominent in the PFC. We also found evidence that WM training restoring some of the aberrant activity, and that neural mechanisms were different in trained and transfer tasks. Importantly, training‐related changes in brain activity were different in the trained and untrained tasks, activity being decreased in the trained task at posttest while in the untrained task an activity increase was observed. This finding might inform future developments of WM training paradigms for ADHD interventions by demonstrating that the near‐transfer may stem from increased brain activity, but it could also make it less durable than task‐specific effects that probably involves automatization. However, given the modest behavioral transfer effects observed here, the present neuroimaging results have at present mostly theoretical value.

## CONFLICT OF INTEREST

None of the authors have any biomedical financial interests or potential conflicts of interest.

## AUTHOR CONTRIBUTIONS

**Juha Salmi:** Experiment design; collection and analysis of the data; manuscript writing and its revision that was commented, complemented, or agreed on by all authors. **Matti Laine:** Experiment design; commented the data‐analyses; selection, recruitment, and diagnosis of the patients. **Anna Soveri:** Experiment design; commented the data‐analyses. **Kimmo Alho:** Experiment design; commented the data‐analyses. **Viljami Salmela:** Experiment design; commented the data‐analyses. **Susanne M. Jaeggi:** Experiment design; commented the data‐analyses. **Pekka Tani:** Experiment design; selection, recruitment, and diagnosis of the patients. **Sami Leppämäki:** Experiment design; selection, recruitment, and diagnosis of the patients. **Anniina Koski:** Selection, recruitment, and diagnosis of the patients.

## Supporting information


**Figure S1** Consolidated Standards Of Reporting Trials (CONSORT) flow diagram illustrating participant recruitment, randomization, and attrition.

**Figure S2** Self‐reported level of motivation and arousal during the training period in each participant in the Experimental group and healthy controls.

**Figure S3** Brain activity in visuospatial versus verbal (digit) n‐back tasks across all participants at each load level (1‐back vs. 0‐back, 2‐back vs. 0‐back, and 3‐back vs. 0‐back). There was no significant brain activity in the opposite contrasts (verbal vs. visuospatial tasks). *Z* = 3.5, corrected *p* < .05.

**Figure S4** Regions of interest used for plotting the data in Figures 2 and 4.

**Table S1** Absolute and relative movements (mean displacements in mm) in each condition of the fMRI measurement.

**Table S2** Task performance in each single n‐back task separately in (untrained) ADHD participants and Healthy controls. *p* represents the group difference.

**Table S3** Comparison between Experimental group and Active control group at pretest. *p* represents the group difference. In education level, 0 is no degree, 4 is high‐school, and 9 is PhD.

**Table S4** Training‐related changes in self‐rated attention and executive function, and task performance. In parenthesis, p‐values from the within group post versus pre comparison.

## Data Availability

We used codes implemented in FSL toolbox. All parameters and details will be available from the authors upon request. As the data will be used also in other papers, publishing parts of it in a public repository will be considered only when the relevant publications have been accepted for publication.
