# Supplementary figures and images for: Working memory training restores aberrant brain activity in adult attention‐deficit hyperactivity disorder
Source: Hum Brain Mapp. 2020 Aug 19;41(17):4876–91. doi: 10.1002/hbm.25164 (PMC7643386; doi:10.1002/hbm.25164)

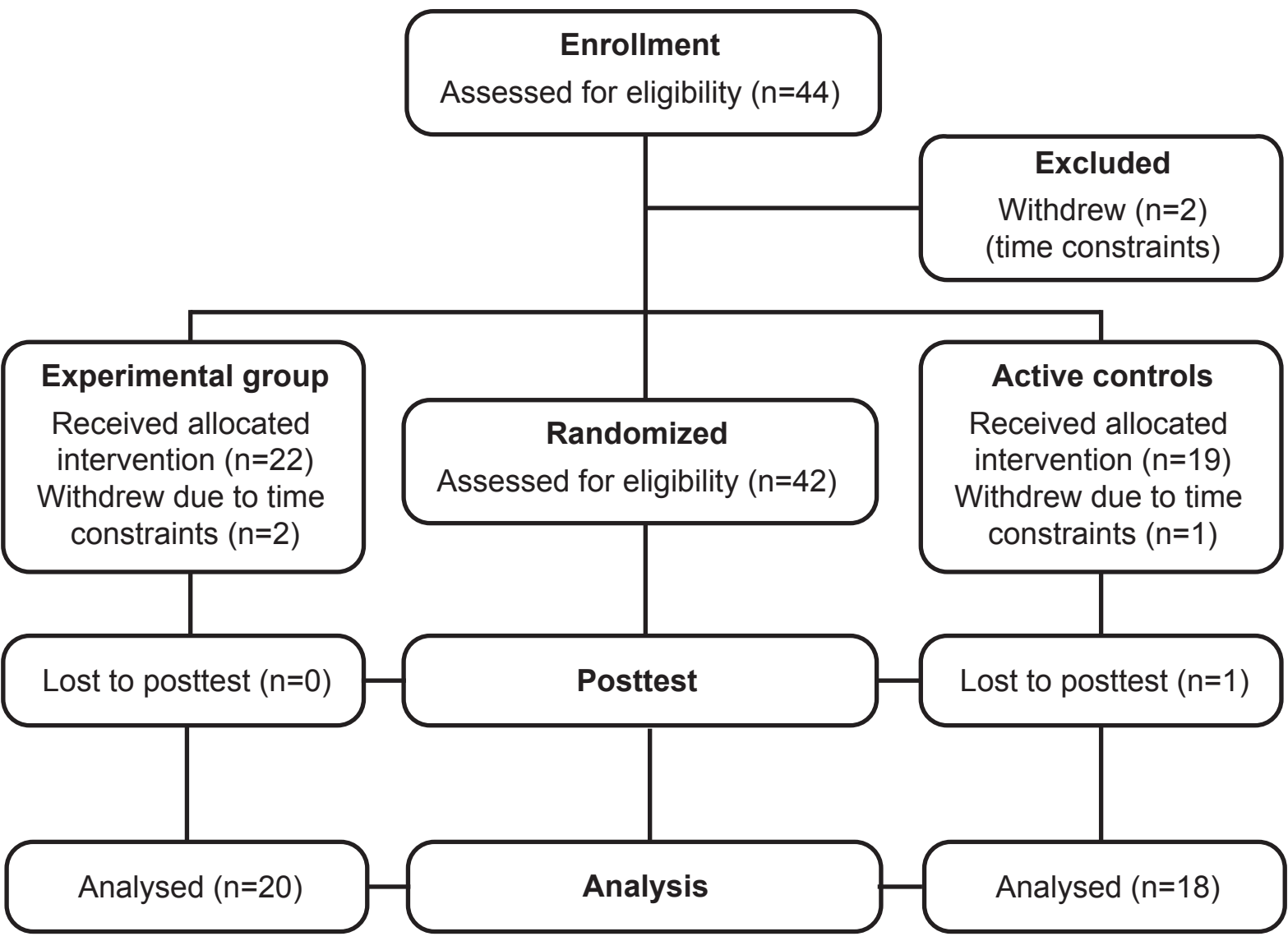

Supplement: Supplementary file 1 — Figure S1 Consolidated Standards Of Reporting Trials (CONSORT) flow diagram illustrating participant recruitment, randomization, and attrition. [file HBM-41-4876-s001.pdf]

# Spatial vs. verbal n-back (all participants)

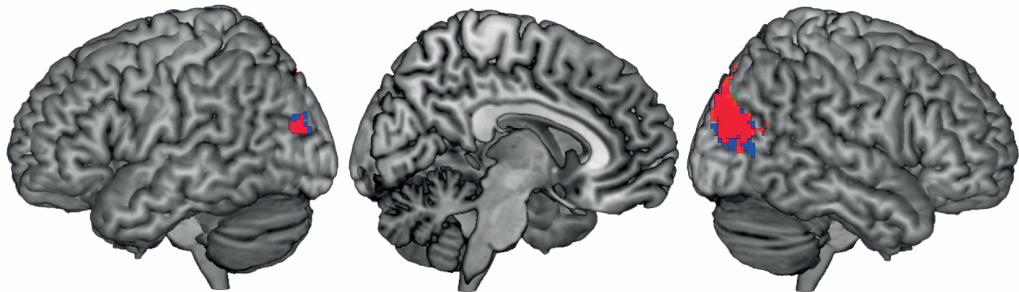

1-back 2-back 3-back

Supplement: Supplementary file 3 — Figure S3 Brain activity in visuospatial versus verbal (digit) n‐back tasks across all participants at each load level (1‐back vs. 0‐back, 2‐back vs. 0‐back, and 3‐back vs. 0‐back). There was no significant brain activity in the opposite contrasts (verbal vs. visuospatial tasks). Z = 3.5, corrected p < .05. [file HBM-41-4876-s003.pdf]

# Region of interest analyses

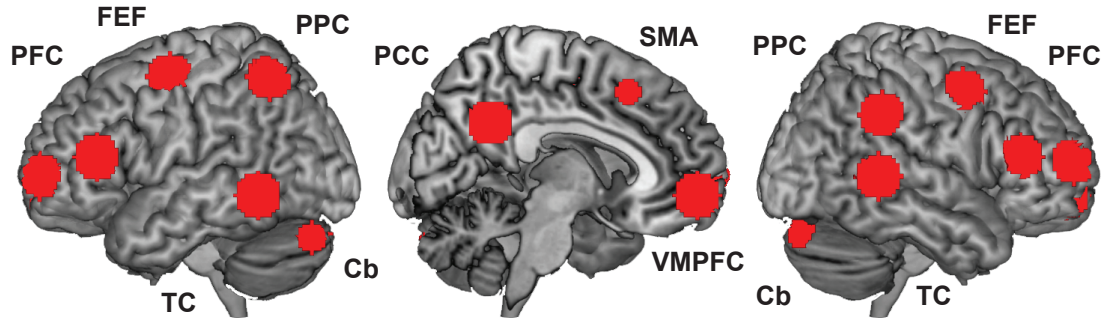

Supplement: Supplementary file 4 — Figure S4 Regions of interest used for plotting the data in Figures 2 and 4. [file HBM-41-4876-s004.pdf]
