# Supplementary material for: Working memory training restores aberrant brain activity in adult attention‐deficit hyperactivity disorder
Source: Hum Brain Mapp. 2020 Aug 19;41(17):4876–91. doi: 10.1002/hbm.25164 (PMC7643386; doi:10.1002/hbm.25164)
Supplement: Supplementary file 2 — Figure S2 Self‐reported level of motivation and arousal during the training period in each participant in the Experimental group and healthy controls. [file HBM-41-4876-s002.pdf]

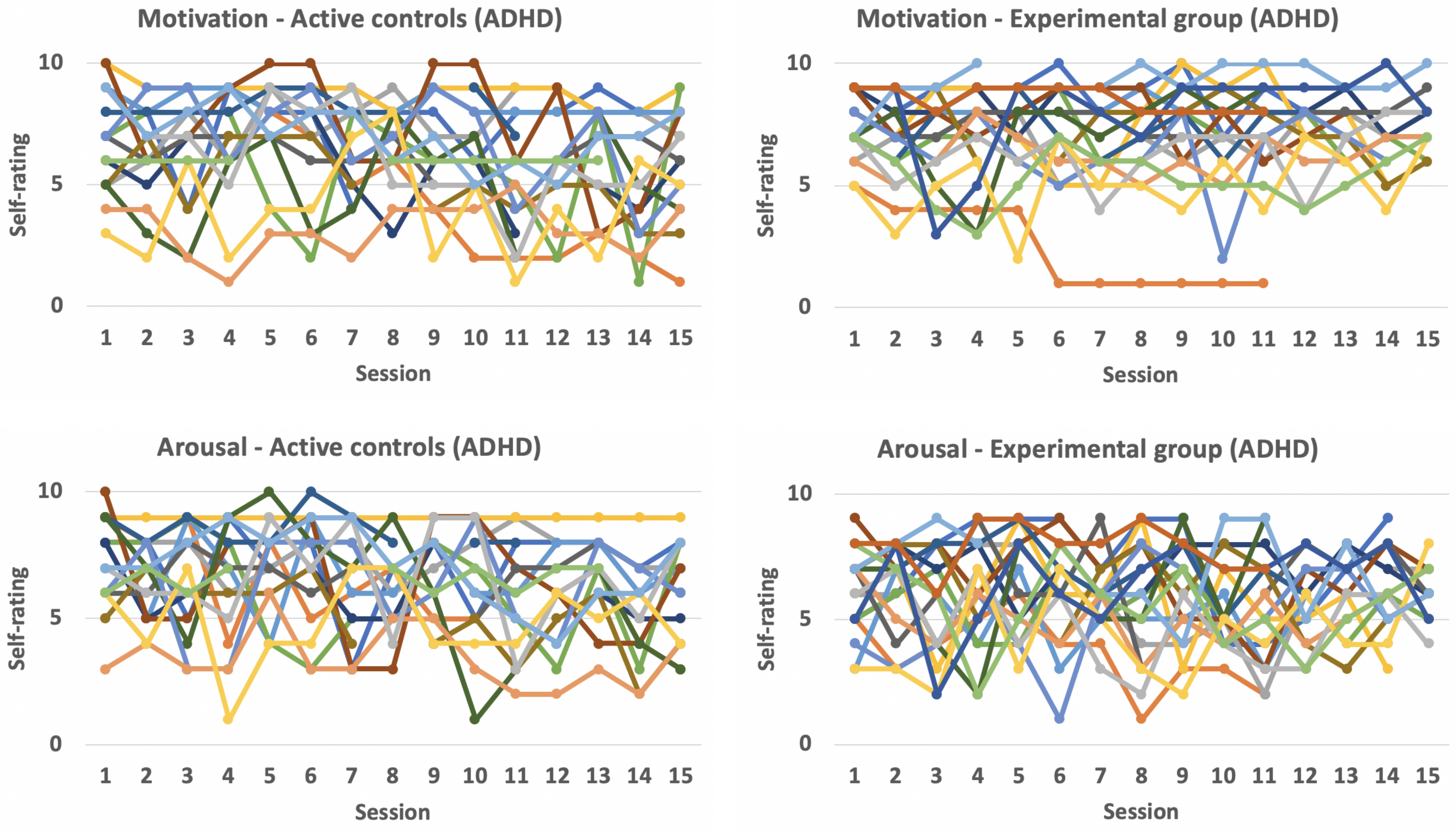
Motivation - Active controls (ADHD)
Motivation - Experimental group (ADHD)
Arousal - Active controls (ADHD)
Arousal - Experimental group (ADHD)
Self-rating
0
5
10
Session
1 2 3 4 5 6 7 8 9 10 11 12 13 14 15
