# Supplementary material for: Working memory training restores aberrant brain activity in adult attention‐deficit hyperactivity disorder
Source: Hum Brain Mapp. 2020 Aug 19;41(17):4876–91. doi: 10.1002/hbm.25164 (PMC7643386; doi:10.1002/hbm.25164)
Supplement: Supplementary file 5 — Table S1 Absolute and relative movements (mean displacements in mm) in each condition of the fMRI measurement. [file HBM-41-4876-s005.doc]

**Supplementary Table 1.** Absolute and relative movements (mean displacements in mm) in each condition of the fMRI measurement.

|  | ADHD | Healthy | Training (pre) | Control (pre) | Training (post) | Control (post) |
| --- | --- | --- | --- | --- | --- | --- |
| **Absolute movement** |  |  |  |  |  |  |
| Spatial n-back | 0.34 (0.36) | 0.32 (0.19) | 0.4 (0.45) | 0.32 (0.26) | 0.31 (0.19) | 0.3 (0.24) |
| Verbal n-back | 0.34 (0.21) | 0.30 (0.17) | 0.44 (0.36) | 0.32 (0.21) | 0.34 (0.18) | 0.32 (0.16) |
| **Relative movement** |  |  |  |  |  |  |
| Spatial n-back | 0.07 (0.06) | 0.08 (0.04) | 0.06 (0.04) | 0.08 (0.07) | 0.07 (0.06) | 0.07 (0.06) |
| Verbal n-back | 0.07 (0.04) | 0.08 (0.03) | 0.06 (0.03) | 0.07 (0.04) | 0.06 (0.04) | 0.07 (0.06) |
