# Supplementary material for: Working memory training restores aberrant brain activity in adult attention‐deficit hyperactivity disorder
Source: Hum Brain Mapp. 2020 Aug 19;41(17):4876–91. doi: 10.1002/hbm.25164 (PMC7643386; doi:10.1002/hbm.25164)
Supplement: Supplementary file 6 — Table S2 Task performance in each single n‐back task separately in (untrained) ADHD participants and Healthy controls. p represents the group difference. [file HBM-41-4876-s006.doc]

| **Supplementary Table 2.** Task performance in each single n-back task separately in (untrained) ADHD participants and Healthy controls. *p* represents the group difference. | | | | |
| --- | --- | --- | --- | --- |
| Variable |  | ADHD (n = 39) | Healthy controls (n = 18) | *p* |
| Single 1-back - spatial | Hitrate (%) | 91 (12) | 93 (7) | 0.47 |
| Single 2-back - spatial | Hitrate (%) | 88 (14) | 87 (9) | 0.72 |
| Single 3-back - spatial | Hitrate (%) | 87 (11) | 88 (9) | 0.82 |
| Single 1-back - verbal | Hitrate (%) | 93 (13) | 95 (7) | 0.54 |
| Single 2-back - verbal | Hitrate (%) | 88 (13) | 88 (11) | 0.91 |
| Single 3-back - verbal | Hitrate (%) | 85 (10) | 87 (6) | 0.35 |
