# Supplementary material for: Working memory training restores aberrant brain activity in adult attention‐deficit hyperactivity disorder
Source: Hum Brain Mapp. 2020 Aug 19;41(17):4876–91. doi: 10.1002/hbm.25164 (PMC7643386; doi:10.1002/hbm.25164)
Supplement: Supplementary file 7 — Table S3 Comparison between Experimental group and Active control group at pretest. p represents the group difference. In education level, 0 is no degree, 4 is high‐school, and 9 is PhD. [file HBM-41-4876-s007.doc]

| **Supplementary Table 3.** Comparison between Experimental group and Active control group at pretest. *p* represents the group difference. In education level, 0 is no degree, 4 is high-school, and 9 is PhD. | | | | |
| --- | --- | --- | --- | --- |
| Variable |  | Training group (n = 20) | Active controls (n = 18) | p |
| **Demographics** |  |  |  |  |
| Age | years | 27.8 (4.9) | 29.3 (5.9) | 0.41 |
| Education | level | 4.7 (2.2) | 4.7 (2.4) | 0.95 |
| Verbal skills (WAIS vocabulary test) | standard score | 4.7 (2.2) | 4.7 (2.4) | 0.95 |
| Non-verbal skills (WAIS matrix reasoning) | standard score | 110 (15.2) | 113 (12.0) | 0.43 |
| BRIEF | sum score | 73.2 (16.6) | 79.9 (23.6) | 0.32 |
| **ADHD symptoms** |  |  |  |  |
| CAADID inattention | sum score | 17.2 (4.1) | 16.2 (4.3) | 0.48 |
| CAADID hyperactivity | sum score | 13.7 (6.6) | 15.0 (5.3) | 0.49 |
| CAADID total | sum score | 30.8 (8.8) | 31.7 (8.3) | 0.7 |
