# Supplementary material for: Working memory training restores aberrant brain activity in adult attention‐deficit hyperactivity disorder
Source: Hum Brain Mapp. 2020 Aug 19;41(17):4876–91. doi: 10.1002/hbm.25164 (PMC7643386; doi:10.1002/hbm.25164)
Supplement: Supplementary file 8 — Table S4 Training‐related changes in self‐rated attention and executive function, and task performance. In parenthesis, p‐values from the within group post versus pre comparison. [file HBM-41-4876-s008.doc]

| **Supplementary Table 4.** Training-related changes in self-rated attention and executive function, and task performance. In parenthesis, p-values from the within group post vs. pre comparison. | | | | | | | | | |
| --- | --- | --- | --- | --- | --- | --- | --- | --- | --- |
|  |  | Healthy controls | Exp. group  pretest | Exp. group  posttest | Exp. group: pre vs. post *p (d)* | Active controls pretest | Active controls posttest | Active controls: pre vs. post *p (d)* | *ANCOVA p (ηp2)* |
| **ADHD symptoms** |  |  |  |  |  |  |  |  |  |
| ASRS-A | Sum score | 7.17 (3.3) | 14.70 (3.73) | 14.00 (3.85) | 0.42 (0.18) | 12.11 (3.16) | 14.53 (4.93) | 0.73 (0.58) | 0.77 (0.003) |
| ASRS-B | Sum score | 14.5 (6.46) | 26.78 (8.01) | 24.65 (6.65) | 0.14 (0.29) | 27.83 (8.08) | 27.75 (8.14) | 0.85 (0.00) | 0.16 (0.06) |
| ASRS total | Sum score | 21.67 (9.41) | 41.48 (11.15) | 38.65 (10.14) | 0.18 (0.27) | 43.06 (11.02) | 40.65 (14.75) | 0.46 (0.18) | 0.78 (0.002) |
| **Cognitive tasks** |  |  |  |  |  |  |  |  |  |
| Dual n-back | Max level | 2.22 (0.55) | 2.11 (0.46) | 3.79 (0.71) | <0.0001 (2.81) | 2.11 (0.66) | 2.58 (0.84) | 0.02 (0.62) | <0.0001 (0.45) |
| Single n-back - spatial | Mean hitrate | 89 (8) | 91 (6) | 89 (7) | 0.30 (0.21) | 90 (8) | 89 (3) | 0.54 (0.16) | 0.77 (0.002) |
| Single n-back – verbal | Mean hitrate | 90 (8) | 91 (6) | 89 (6) | 0.32 (0.20) | 89 (9) | 90 (3) | 0.33 (0.21) | 0.68 (0.08) |
| Running Memory - spatial | Lists correct | 4.22 (1.99) | 3.1 (2.05) | 4.7 (1.79) | <0.001 (0.83) | 3.4 (2.19) | 3.8 (2.24) | 0.72 (0.18) | 0.01 (0.12) |
| Running Memory - verbal | Lists correct | 3.28 (1.93) | 3.4 (1.7) | 3.42 (1.47) | 0.9 (0.01) | 3.3 (1.92) | 3.85 (2.13) | 0.12 (0.27) | 0.20 (0.03) |
| Digit Span | Sum score | 13.67 (3.03) | 10.8 (2.65) | 12.3 (2.68) | <0.0001 (0.56) | 12.11 (3.16) | 12.72 (2.47) | 0.26 (0.22) | 0.37 (0.02) |
| CPT – omission errors | Error score | 1.28 (1.64) | 1.81 (3.52) | 1.58 (2.61) | 0.65 (0.07) | 2.96 (4.32) | 0.65 (1.23) | 0.03 (0.73) | 0.13 (0.06) |
| CPT – commission errors | Error score | 9.28 (4.70) | 18.33 (8.18) | 9.32 (7.32) | <0.0001 (1.16) | 16.00 (5.84) | 7.6 (6.22) | <0.0001 (1.39) | 0.74 (0.003) |
